# Analyzing the similarity of samples and genes by MG-PCC algorithm, t-SNE-SS and t-SNE-SG maps

**DOI:** 10.1186/s12859-018-2495-5

**Published:** 2018-12-17

**Authors:** Xingang Jia, Qiuhong Han, Zuhong Lu

**Affiliations:** 1 School of Mathematics, Southeast University, Nanjing, 210096 People’s Republic of China; 2 Department of Mathematics, Nanjing Forestry University, Nanjing, 210037 People’s Republic of China; 3 State Key Laboratory of Bioelectronics, School of Biological Science and Medical Engineering, Southeast University, Nanjing, 210096 People’s Republic of China

**Keywords:** PCC, MG-PCC, t-SNE-SSP, t-SNE-SGI, A-value

## Abstract

**Background:**

For analyzing these gene expression data sets under different samples, clustering and visualizing samples and genes are important methods. However, it is difficult to integrate clustering and visualizing techniques when the similarities of samples and genes are defined by PCC(Person correlation coefficient) measure.

**Results:**

Here, for rare samples of gene expression data sets, we use MG-PCC (mini-groups that are defined by PCC) algorithm to divide them into mini-groups, and use t-SNE-SSP maps to display these mini-groups, where the idea of MG-PCC algorithm is that the nearest neighbors should be in the same mini-groups, t-SNE-SSP map is selected from a series of t-SNE(t-statistic Stochastic Neighbor Embedding) maps of standardized samples, and these t-SNE maps have different perplexity parameter. Moreover, for PCC clusters of mass genes, they are displayed by t-SNE-SGI map, where t-SNE-SGI map is selected from a series of t-SNE maps of standardized genes, and these t-SNE maps have different initialization dimensions. Here, t-SNE-SSP and t-SNE-SGI maps are selected by A-value, where A-value is modeled from areas of clustering projections, and t-SNE-SSP and t-SNE-SGI maps are such t-SNE map that has the smallest A-value.

**Conclusions:**

From the analysis of cancer gene expression data sets, we demonstrate that MG-PCC algorithm is able to put tumor and normal samples into their respective mini-groups, and t-SNE-SSP(or t-SNE-SGI) maps are able to display the relationships between mini-groups(or PCC clusters) clearly. Furthermore, t-SNE-SS(m)(or t-SNE-SG(n)) maps are able to construct independent tree diagrams of the nearest sample(or gene) neighbors, where each tree diagram is corresponding to a mini-group of samples(or genes).

**Electronic supplementary material:**

The online version of this article (10.1186/s12859-018-2495-5) contains supplementary material, which is available to authorized users.

## Background

With the rapid development of high-throughput biotechnologies, we were easily able to collect a large amount of gene expression data with many subjects of biology or medicine [1]. Here, we aimed at these gene expression data sets that came from tumoral and normal samples, where these data sets were often characterized by mass genes but with relatively small amounts of samples, their rows were corresponding to genes, and columns were representing samples [2]. For these gene expression data sets, they usually incorporated several thousands of probes associated with more and less relevance for cancers [3]. Thus, the filtering approaches applied to each probe before data analysis, with the aim to find differentially expressed genes, such as T-statistics, Significance Analysis, Adaptive Ranking, Combined Adaptive Ranking and Two-way Clustering [4, 5]. For samples of gene expression data sets, a major challenge was how to resolve their subtypes, and compare in different diseased states [4, 6]. Much work had been done on exploratory subtypes of cancers, such as Hierarchical clustering, K-means, penalised likelihood methods and the random forest [7, 8]. Moreover, to determine the intrinsic dimensionality of genes, the clustering analysis was used to search for patterns and group genes into expression clusters that provided additional insight into the biological function and relevance of genes that showed different expressions [9–13]. Furthermore, to display classification of genes(or samples) in a meaningful way for exploration, presentation, and comprehension in diseased states and normal differentiation, many dimension reduction techniques were used to embed high-dimensional data for visualization in 2D(two dimensional) spaces [14–17], and had been successful in complementing clusters of Euclidean distance [14], such as Hierarchical clustering dendrograms, PCA(principal component analysis), t-SNE, heat maps, and network graphs [14–18].

For samples of gene expression data sets, their dimensionality often resulted in their different types to be isometric by Euclidean distance [9]. Thus, in the process of samples and genes clustering analysis, PCC commonly used also [10, 12, 13]. The simplest way to think about PCC was to plot curves of two genes, with PCC telling us how similar the shapes of their two curves were. But for PCC clusters of gene expression data, many projection techniques gave them poor visualizations usually [16]. To efficiently map clusters of PCC, PCC had been defined by transformed genes, such as PCCF(PCC of F-points) and PCC-MCP(PCC of multiple-cumulative probabilities) [19, 20]. Moreover, PCA-F and t-SNE-MCP-O gave good visualizations for clusters of PCCF and PCC-MCP, respectively. However, for PCC clusters of the original gene expression points, PCA-F and t-SNE-MCP-O gave them poor visualizations also [19, 20].

Here, for samples of gene expression data sets, we used MG-PCC algorithm to divide them into different mini-groups, where the similarities of samples were defined by PCC measure, and the idea of MG-PCC algorithm is that the nearest neighbors should be in the same mini-groups. That is, for any sample of a mini-group, its nearest neighbor was in the mini-group also. Moreover, we used t-SNE-SSP maps to display the relationships of mini-groups, where t-SNE-SSP map was selected from a series of t-SNE maps of standardized samples, these t-SNE maps had different perplexity parameter, and the initialization dimensions of these t-SNE maps were thirty. In t-SNE, the perplexity might be viewed as a knob that sets the number of effective nearest neighbors. It was comparable with the number of nearest neighbors that was employed in many manifold learners [21, 22].

Furthermore, for gene clusters that were generated from PCC, we attempted to use t-SNE-SGI maps to display them, where t-SNE-SGI maps were selected from a series of t-SNE maps of standardized genes. Compared to t-SNE-SSP maps, t-SNE-SGI map was selected from these t-SNE maps that had the same perplexity parameter, but different initialization dimensions, where the perplexity parameter of these t-SNE maps were the dimensions of genes. In fact, for gene expression data sets under different samples, their genes were mass and dense, and the performance of t-SNE with these data sets required a larger perplexity.

Here, we used A-value to select the t-SNE-SSP and t-SNE-SGI maps, where A-value was modeled from areas of clustering projections, and a t-SNE map was selected as t-SNE-SSP(or t-SNE-SGI) if its A-value was the smallest compared to others. Furthermore, for clusters with different clustering number, their t-SNE-SGI maps might come from the different t-SNE maps.

To evaluate the reliability of the MG-PCC and t-SNE-SSP, we applied them to gene expression data sets of lung cancers [23, 24]. Results showed that MG-PCC algorithm was able to put tumor and normal samples into their respective mini-groups, and t-SNE-SSP maps gave these mini-groups clear boundaries also, which helped us to mine the subtypes of cancers. Moreover, for PCC clusters of genes, t-SNE-SGI maps gave them better visualizations compared to t-SNE of the original and normalized genes, which made clustering and visualizing techniques better integration. Furthermore, for the nearest sample(or gene) neighbors, t-SNE-SS(m)(or t-SNE-SG(n)) maps were able to give them independent tree diagrams, where each tree diagram was corresponding to a mini-group of samples(or genes).

## Materials and methods

***Data and data source***

The first data set GDS3837 provides insight into potential prognostic biomarkers and therapeutic targets for non-small cell lung carcinoma, where it has 54674 genes, 60 normal and 60 tumor samples that are taken from nonsmoking females [23, 24]. The second data set GDS3257 provides insight into the molecular basis of lung carcinogenesis induced by smoking, where it has a total of 22283 genes, and contains 107 samples that are taken from former, current and never smokers [23, 24], where GDS3837 and GDS3257 can be downloaded from NCBI’s GEO Database.

Here, we firstly use GDS3257 and GDS3837 to construct 5 matrixes, where *A*_*k*_(k =1, 2, 3, 4 and 5) is the *k*-th matrix, the *i*-th row of *A*_*k*_ represents the *i*-th gene, the *j*-th column represents the *j*-th sample, genes of *A*_*k*_ are filtered by T-test(Hypothesis testing for the difference in means of two types of samples), and the detail of *A*_*k*_ is summarized in Table 1. Then, 5 sample data sets are constructed by *A*_*k*_, where data-k(k =1, 2, 3, 4 and 5) is the *k*-th sample data set, and data-k is transposed matrix of *A*_*k*_. And then, 5 gene data sets are constructed by *A*_*k*_, where data-(k+5)(k =1, 2, 3, 4 and 5) is the *k*-th gene data set, and data-(k+5) only contains tumor samples of *A*_*k*_. That is, *A*_*k*_ is represented by (*B*_*k*_,*C*_*k*_), and data-(k+5)(k =1, 2, 3, 4 and 5) is *B*_*k*_, where *B*_*k*_ and *C*_*k*_ contains tumor and normal samples, respectively.

**Table 1:** The details of *A*_*k*_(k=1, 2, 3, 4 and 5)

*A*_*k*_	NO. of	NO. of	Tumor	Control	*P*-value
	genes	samples	samples(*B*_*k*_)	samples(*C*_*k*_)	*P*-value
*A*_1_	1355	31	16 TN-smokers	15 NN-smokers	< 10^−5^
			(GDS3257)	(GDS3257)	
*A*_2_	1129	36	18 TF-smokers	18 NF-smokers	< 10^−5^
			(GDS3257)	(GDS3257)	
*A*_3_	2055	40	24 TC-smokers	16 NC-smokers	< 10^−5^
			(GDS3257)	(GDS3257)	
*A*_4_	817	76	18 TF-smokers,	18NF-smokers	< 10^−5^
			24 TC-smokers	16NC-smokers	< 10^−5^
			(GDS3257)	(GDS3257)	∗
*A*_5_	1739	120	60 TN-smokers	60 NN-smokers	< 10^−12^
			(GDS3837)	(GDS3837)	

TN-smokers: tumor never-smokers; TF-smokers: tumor former-smokers; TC-smokers: tumor current-smokers; NN-smokers: normal never-smokers; NF-smokers: normal former-smokers; NC-smokers: normal current-smokers. ∗ was that *P*-value of the mixing samples was less than < 10^−5^ also

### Methods

Here, we use *X*_*i*_ to represent the *i*-th sample of data-k(k =1, 2, 3, 4 and 5), and *Y*_*j*_ to represent the *j*-th gene of data-(k+5). That is, *X*_*i*_ is the *i*-th row of data-k(k =1, 2, 3, 4 and 5), and *Y*_*j*_ is the *j*-th row of data-(k+5), where

1 $$ \left\{ \begin{aligned} X_{i}=\{x_{i1},x_{i2},\cdots,x_{im}\},\\ Y_{j}=\{y_{j1},y_{j2},\cdots,y_{jn}\}. \end{aligned} \right.  $$

#### S-points

Here, *X*_*i*_ and *Y*_*j*_ are standardized into *SS*_*i*_ and *SG*_*j*_, where *SS*_*i*_ and *SG*_*j*_ are called as S-sample and S-gene of *X*_*i*_ and *Y*_*j*_ respectively, and

2 $$ \left\{ \begin{aligned} \!\!SS_{i}&\,=\,\{ss_{i1},ss_{i2},\cdots,ss_{im}\},ss_{it}\,=\,\!\frac{x_{it}-EX_{i}}{\sqrt{DX_{i}}},\!~t\,=\,1,2,\cdots,m, \\ \!\!EX_{i}&=\frac{\sum\limits_{l=1}^{m }x_{il}}{m}, DX_{i}=\frac{\sum\limits_{l=1}^{m }(x_{il}-EX_{i})^{2}}{m-1}.\\ \end{aligned} \right.  $$

#### MG-PCC algorithm

Here, for *X*_*j*1_ and *X*_*j*2_, they are used to construct the first mini-group, where

3 $$\begin{array}{@{}rcl@{}} \rho(X_{j1},X_{j2})=\max\limits_{1\leq i< j \leq u} \{\rho(X_{i},X_{j})\}, \end{array} $$

*ρ*(*X*_*i*_,*X*_*j*_) is PCC between *X*_*i*_ and *X*_*j*_, and *u* is the number of samples. For *X*_*j*3_, it is put into the first mini-group if it satisfies

4 $$\begin{array}{@{}rcl@{}} \max\{\rho(X_{j3},X_{j1}),\rho(X_{j3},X_{j2})\}=\max\limits_{1\leq i \leq u} \{\rho(X_{i},X_{j3})\}. \end{array} $$

When the first mini-group contains (*t*−1)(*t*>3) samples, *X*_*jt*_ is put into the first mini-group if it satisfies

5 $$\begin{array}{@{}rcl@{}} \max\limits_{1\leq i \leq u, i\neq jt} \{\rho(X_{jt},X_{i})\}=& \max\{\rho(X_{jt},X_{j1}),\rho(X_{jt},X_{j2}),\cdots,\\&\rho(X_{jt},X_{j(t-1)})\}, \end{array} $$

where *X*_*j*1_,*X*_*j*2_,⋯,*X*_*j*(*t*−1)_ belong to the first mini-group. Continuously, the first mini-group is completely built until no sample satisfies Eq. (5).

The remaining samples repeat above step until all mini-groups are completely built. For a mini-group, it is completely built if no sample satisfies Eqs. (4) or (5), that is, a mini-group contains two genes at least. Similarly, MG-Euclidean algorithm can be used to construct mini-group also, where the algorithm uses Euclidean distance to define the similarities of samples.

#### The A-value

For samples of each mini-group, we plot the boundary of their projections by a closed line, where the closed line is called as boundary-line of the mini-group, the boundary-line forms a convex hull of their projections, and the area of the convex hull is called as A-value of the mini-group. Here, we use A-value to describe the consistency between samples and their projections, where

6 $$\begin{array}{*{20}l} &A=\frac{{\sum\nolimits}_{i=1}^{v}a_{i}}{a}, \end{array} $$

*a*_*i*_ is A-value of the *i*-th mini-group, *a* is A-value of the data set, *v* is the number of mini-groups.

In general, for adjacent mini-groups, there is often some overlap for their convex hulls. Thus, A-value is smaller, the consistency between points and projections is more valid.

#### The t-SNE-SSP and t-SNE-SGI

Using t-SNE requires tuning some parameters, notably the perplexity and initialization dimension. Although t-SNE results are robust to the settings of parameters, in practice, we still have to interactively choose parameters by visually comparing results under multiple settings. For mini-groups and clusters of samples that are generated from PCC, we empirically validate that t-SNE maps of the standardized samples with an appropriate perplexity can clearly display them, where the initialization dimension of these t-SNE maps is thirty. But for PCC clusters of genes, t-SNE maps of S-genes with an appropriate initialization dimension can give them good visualizations, where the perplexity parameter of these t-SNE maps is the dimensions of genes.

Here, for mini-groups and clusters of samples that are generated from PCC, their t-SNE-SSP map is selected from a series of t-SNE-SS(k) maps by A-value, where t-SNE-SS(k) is t-SNE map of the standardized samples, its initialization dimensions are thirty, its perplexity parameter is *k*, and the value of *k* ranges from 3 to 30. That is, for t-SNE-SS(t), it is selected as t-SNE-SSP if its A-value is the smallest compared to other t-SNE-SS(k). Similarly, for PCC clusters of genes, their t-SNE-SGI map is selected from a series of t-SNE-SG(i) maps by A-value also, where t-SNE-SG(i) is t-SNE map of S-genes, its perplexity parameter of these t-SNE maps is the dimensions of genes, its initialization dimensions is *i*, the value of *i* ranges from 3 to the dimensions of genes.

#### Accuracy, F-Measure, RI and NMI

For t-SNE maps, since they are able to give good visualizations for clusters of Euclidean distance, they can be successful in complementing these PCC clusters that are relative consistency with Euclidean ones. Here, we use Accuracy, F-Measure, RI(Rand index) and NMI(Normalized mutual information) [25, 26] (http://nlp.stanford.edu/IR-book/html/htmledition/evaluation-of-clustering-1.html) to evaluate the consistency of clusters between PCC and Euclidean distance, where clusters of Euclidean distance are seen as the gold standard of genes. In general, Accuracy is a simple and transparent evaluation measure, RI penalizes both false positive and false negative decisions during clustering, F-Measure in addition supports differential weighting of these two types of errors, and NMI can be information theoretically interpreted, where the detailed explanation of these four criteria are explained see in [25, 26], (http://nlp.stanford.edu/IR-book/html/htmledition/evaluation-of-clustering-1.html) and their matlab codes are available at Additional file 1. Furthermore, the higher value of these four criteria means that the more consistency of clusters between PCC and Euclidean distance.

## Results

### The reliability of mini-groups

To test the reliability of mini-groups, we applied MG-PCC and MG-Euclidean algorithms to 5 sample data sets, where MG-Euclidean applied to the standardized samples, O-samples and N-samples simultaneously, O-samples and N-samples were the original and normalized samples respectively, and results of mini-groups were summarized in Table 2. Here, for a mini-group, it was regarded as tumor group if its tumor samples were more than normal ones, otherwise, it was a normal one. Moreover, for a tumor(or normal) sample, it was misjudged if it was put into a normal(or tumor) group. For MG-PCC and MG-Euclidean, Table 2 showed that they correctly judged all samples of data-1 and data-3, and only a few samples of data-2, data-4 and data-5 were misjudged. For instance, only 2 normal and 2 tumor samples of data-5 were misjudged by MG-PCC algorithm, where data-5 contained 60 normal and 60 tumor samples. That is, MG-PCC algorithm was able to put tumor and normal samples into their respective mini-groups, which could help us to compare in different diseased states and normal samples.

**Table 2:** Statistics of the mini-groups of 5 sample data sets

data-i	Algorithm: MG-	NO. of mini-groups	NO. of misjudged tumor samples	NO. of misjudged normal samples
data-1	PCC	4	0	0
	Euclidean-1	3	0	0
	Euclidean-2	4	0	0
	Euclidean-3	4	0	0
data-2	PCC	7	0	3
	Euclidean-1	6	0	3
	Euclidean-2	6	0	2
	Euclidean-3	7	0	0
data-3	PCC	5	0	0
	Euclidean-1	5	0	0
	Euclidean-2	4	0	0
	Euclidean-3	6	0	0
data-4	PCC	13	0	1
	Euclidean-1	13	0	1
	Euclidean-2	12	0	1
	Euclidean-3	10	0	0
data-5	PCC	23	2	2
	Euclidean-1	23	2	3
	Euclidean-2	24	2	2
	Euclidean-3	24	0	4

Euclidean-1: Euclidean distance of O-samples; Euclidean-2: Euclidean distance of the standardized samples; Euclidean-3: Euclidean distance of N-samples

### The clustering feature of S-genes

Here, for data-6, 7, 8, 9 and 10, their S-genes, N-genes and O-genes were divided into clusters by K-means with Euclidean distance and PCC, respectively, where N-genes and O-genes were the original and normalized genes, respectively. Then, Accuracy, F-Measure, RI and NMI were used to demonstrate the consistency of clusters between PCC and Euclidean distance, where clusters of Euclidean distance were seen as the gold standard of genes. For comparison, Accuracy, F-Measure, RI and NMI of these PCC clusters were summarized in Table 3. For clusters of any data set, Table 3 showed that their Accuracy, F-Measure, RI and NMI of S-genes were far more than N-genes and O-genes. That is, for S-genes, their clusters of PCC and Euclidean were more consistent compared to O-genes and N-genes.

**Table 3:** Statistics of Accuracy, F-Measure, RI and NMI of S-genes, N-genes and O-genes

Data	NO. of clusters	Genes	Accuracy	F-Measure	RI	NMI
data-6	6	S-genes	0.956	0.956	0.323	0.888
	7	S-genes	0.794	0.788	0.092	0.672
	8	S-genes	0.803	0.799	0.094	0.660
	6	N-genes	0.321	0.267	-0.084	0.422
	6	O-genes	0.318	0.263	-0.081	0.240
data-7	6	S-genes	0.901	0.897	0.237	0.744
	7	S-genes	0.825	0.814	0.119	0.742
	8	S-genes	0.811	0.813	0.096	0.683
	6	N-genes	0.305	0.271	-0.079	0.487
	6	O-genes	0.308	0.264	-0.079	0.284
data-8	5	S-genes	0.956	0.956	0.293	0.911
	6	S-genes	0.947	0.947	0.257	0.782
	7	S-genes	0.937	0.937	0.181	0.733
	8	S-genes	0.884	0.882	0.137	0.718
	6	N-genes	0.275	0.251	-0.084	0.540
	6	O-genes	0.281	0.249	-0.089	0.340
data-9	5	S-genes	0.977	0.977	0.503	0.842
	6	S-genes	0.827	0.830	0.126	0.814
	7	S-genes	0.947	0.947	0.374	0.741
	8	S-genes	0.962	0.962	0.575	0.693
	9	S-genes	0.893	0.891	0.224	0.692
	8	N-genes	0.258	0.245	-0.070	0.442
	8	O-genes	0.229	0.225	-0.086	0.3043
data-10	3	S-genes	0.901	0.897	0.132	0.902
	4	S-genes	0.829	0.824	0.083	0.807
	5	S-genes	0.716	0.669	0.054	0.601
	6	S-genes	0.779	0.776	0.115	0.714
	7	S-genes	0.895	0.894	0.092	0.683
	8	S-genes	0.593	0.623	-0.008	0.674
	3	N-genes	0.299	0.290	-0.087	0.637
	3	O-genes	0.277	0.203	-0.081	0.088

In general, for data with a normal distribution, the patterns revealed by the clusters under PCC and Euclidean roughly agreed with each other. But for O-genes and N-genes of complex gene expression data sets, results showed that their PCC and Euclidean clusters had significant differences.

### The reliability of A-value

**Fig. 1:**
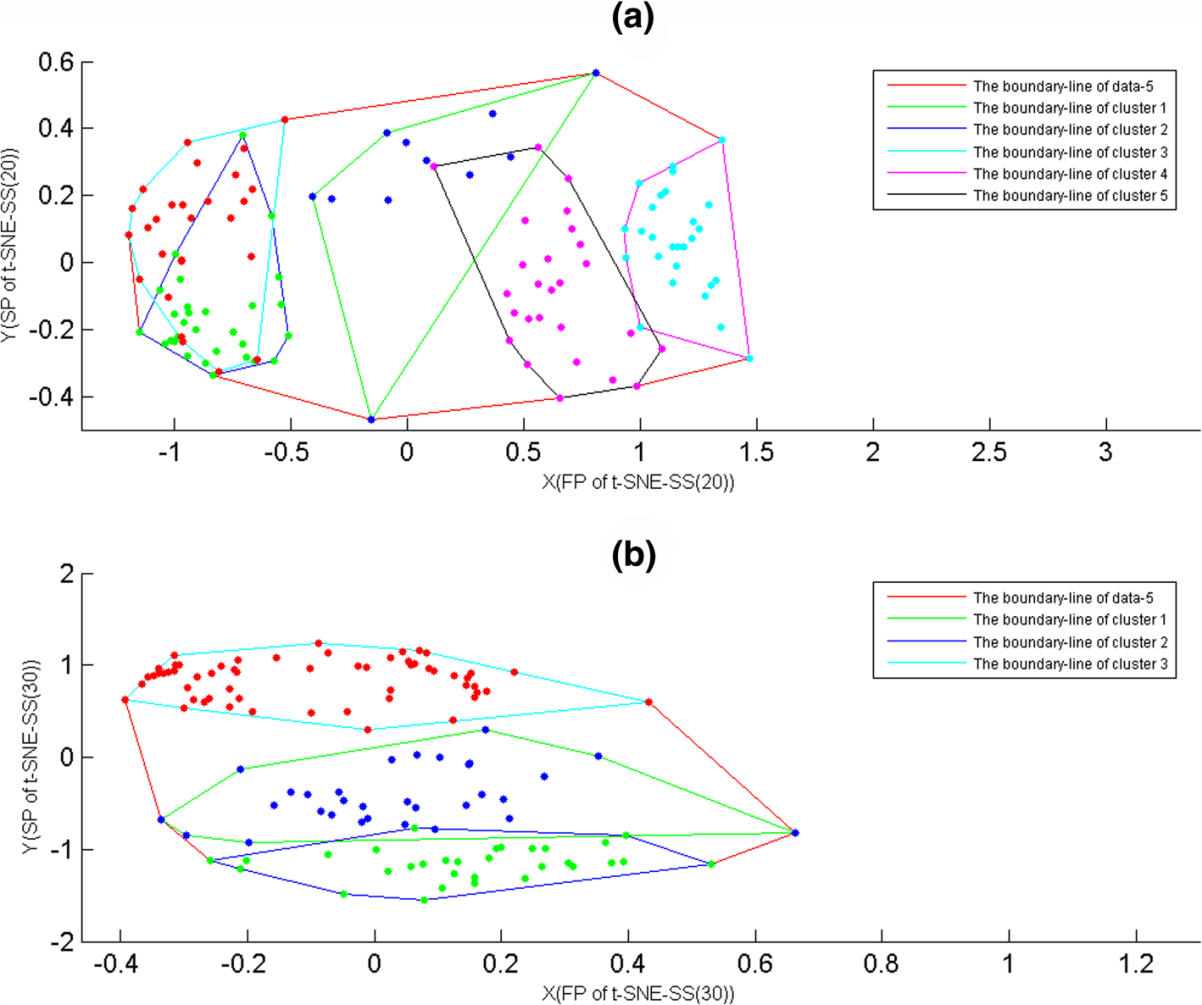
The boundary-lines of data-5. The samples of data-5 were divided into 5 and 3 clusters by K-means with PCC. **a** The boundary-lines of 5 clusters. The X-axis represented the first projections(FP) of t-SNE-SS(20). The Y-axis represented the second projections(SP) of t-SNE-SS(20). **b** The boundary-lines of 3 clusters. The X-axis represented the first projections(FP) of t-SNE-SS(30). The Y-axis represented the second projections(SP) of t-SNE-SS(30)

Here, we used clusters of data-5 to exemplify that A-value was able to quantify the validity of projecting maps, where samples of data-5 were divided into 5 and 3 clusters by K-means with PCC. For 5 and 3 clusters of data-5, they were displayed on t-SNE-SS(20) and t-SNE-SS(30) maps (Fig. 1(a) and 1(b)) respectively, and the boundary-lines of clustering projections were showed on Fig. 1(a) and 1(b) also. For t-SNE-SS(30) map of data-5, it gave good visualizations for 3 clusters (Fig. 1(b)), but t-SNE-SS(20) had slightly intermixing for 5 clusters (Fig. 1(a)). Moreover, for the boundary-lines of t-SNE-SS projections, 5 clusters had more significant overlaps than ones of 3 clusters, while A-value increased with area of overlap. That is, A-value was larger, the consistency between points and projections was more invalid.

### Selecting t-SNE-SSP maps by A-value

**Fig. 2:**
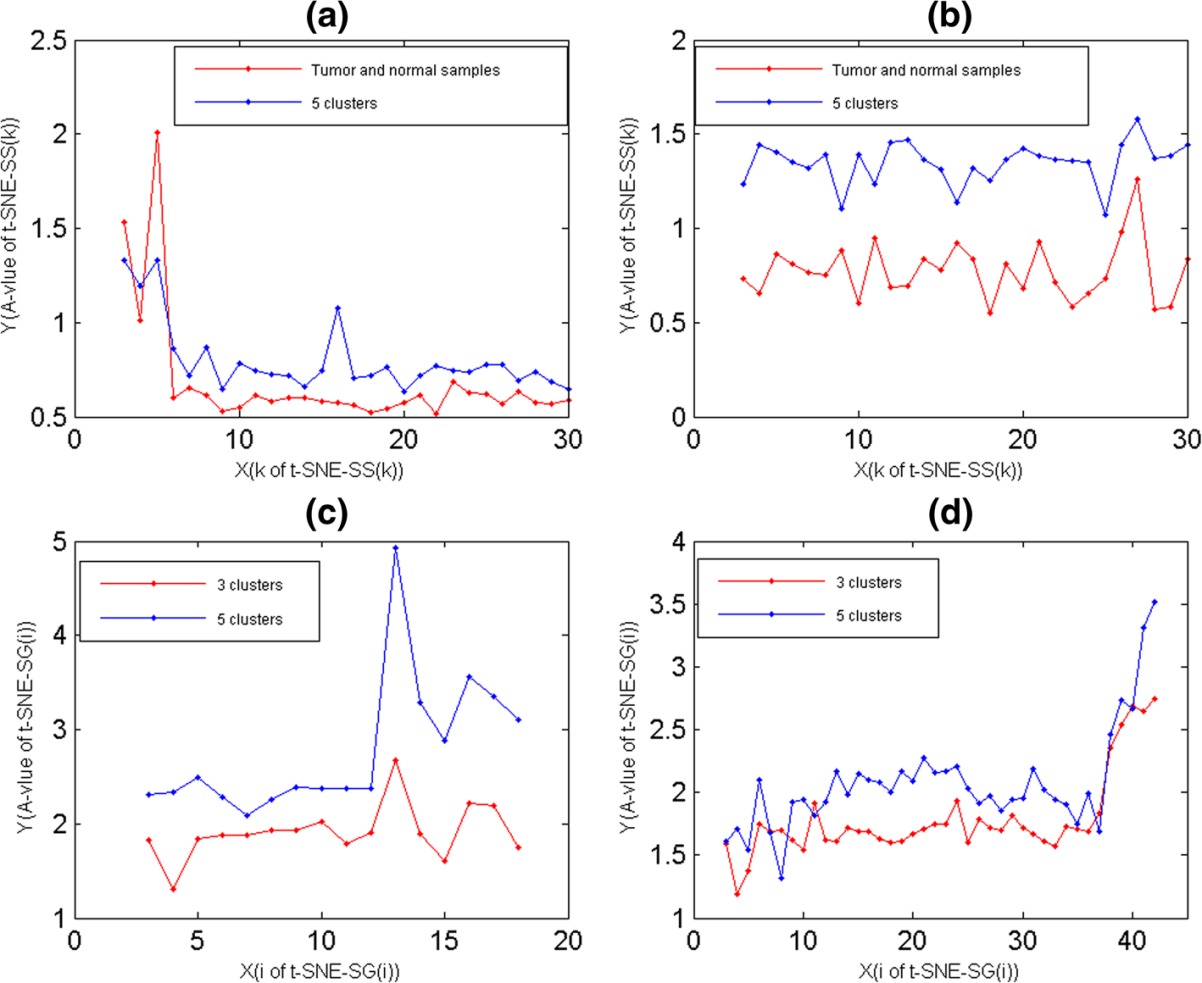
The A-values of t-SNE-SS(k) and t-SNE-SG(i) maps. **a** The A-value of t-SNE-SS(k) maps of data-4. The A-value of 5 PCC clusters were displayed by blue lines, and ones of normal and tumor samples were displayed by red lines. **b** The A-value of t-SNE-SS(k) maps of data-5. The A-value of 5 PCC clusters were displayed by blue lines, and ones of normal and tumor samples were displayed by red lines. **c** The A-value of t-SNE-SG(i) maps of data-7. The A-value of 5 PCC clusters were displayed by blue lines, and ones of 3 PCC clusters were displayed by red lines. **d** The A-value of t-SNE-SG(i) maps of data-9, The A-value of 5 PCC clusters were displayed by blue lines, and ones of 3 PCC clusters were displayed by red lines

Here, for data-4 and data-5, their O-samples were divided into 5 clusters by K-means with PCC, respectively. Then, for clustering results of data-4 and data-5, their A-values of different t-SNE-SS(k) maps were obtained by Eq. (6), where these A-values were showed by blue lines in Fig. 2 (a) and (b), respectively. For different t-SNE-SS(k) maps, Fig. 2 (a) and (b) showed that their A-values had significant difference, A-values of t-SNE-SS(20) and t-SNE-SS(25) were the minimum for 5 clusters of data-4 and data-5, respectively. That is, t-SNE-SS(20) and t-SNE-SS(25) were the optimal 2D maps for 5 clusters of data-4 and data-5, respectively.

Moreover, for 2 clusters of data-4 and data-5 according to normal and tumor samples, their A-values of different t-SNE-SS(k) maps were showed in Fig. 2 (a) and (b) also, where these A-values were showed by red lines. From Fig. 2 (a) and (b), t-SNE-SS(30) was not the optimal 2D maps for any data set also. For 2 clusters of data-5, A-values of its t-SNE-SS(30) was 0.58779, while t-SNE-SS(18) was 0.52373. That is, t-SNE-SS(18) was more appropriate for displaying 5 clusters of data-5.

### Selecting t-SNE-SGI maps by A-value

Here, for gene clusters of data-7 and data-9, their A-values of t-SNE-SG(i) maps were showed in Fig. 2 (c) and (d) respectively, where O-genes of each data set were divided into 3 and 5 clusters by K-means with PCC. Figure 2 (c) and (d) showed that t-SNE-SG(m) maps were not the optimal 2D maps for any clustering result, t-SNE-SG(4) maps were t-SNE-SGI maps of 3 clusters of data-7 and data-9, t-SNE-SG(7) map was t-SNE-SGI maps of 5 clusters of data-7, and t-SNE-SG(8) map was t-SNE-SGI maps of 5 clusters of data-9, respectively.

By Accuracy, F-Measure, RI and NMI, we demonstrated that PCC and Euclidean clusters of S-genes were relative consistent, which enabled t-SNE-SG(i) maps for displaying PCC clusters. But for t-SNE map with the randomly choosing parameters, it could give poor visualization for PCC clusters, which could lead to misinterpretation of clusters. Here, we used A-value to quantify the quality of t-SNE-SG(i) maps, which enabled t-SNE-SGI maps to project genes of the same clusters together, and neighbor clusters in adjacent regions.

### The biological reliability of t-SNE-SSP maps

**Fig. 3:**
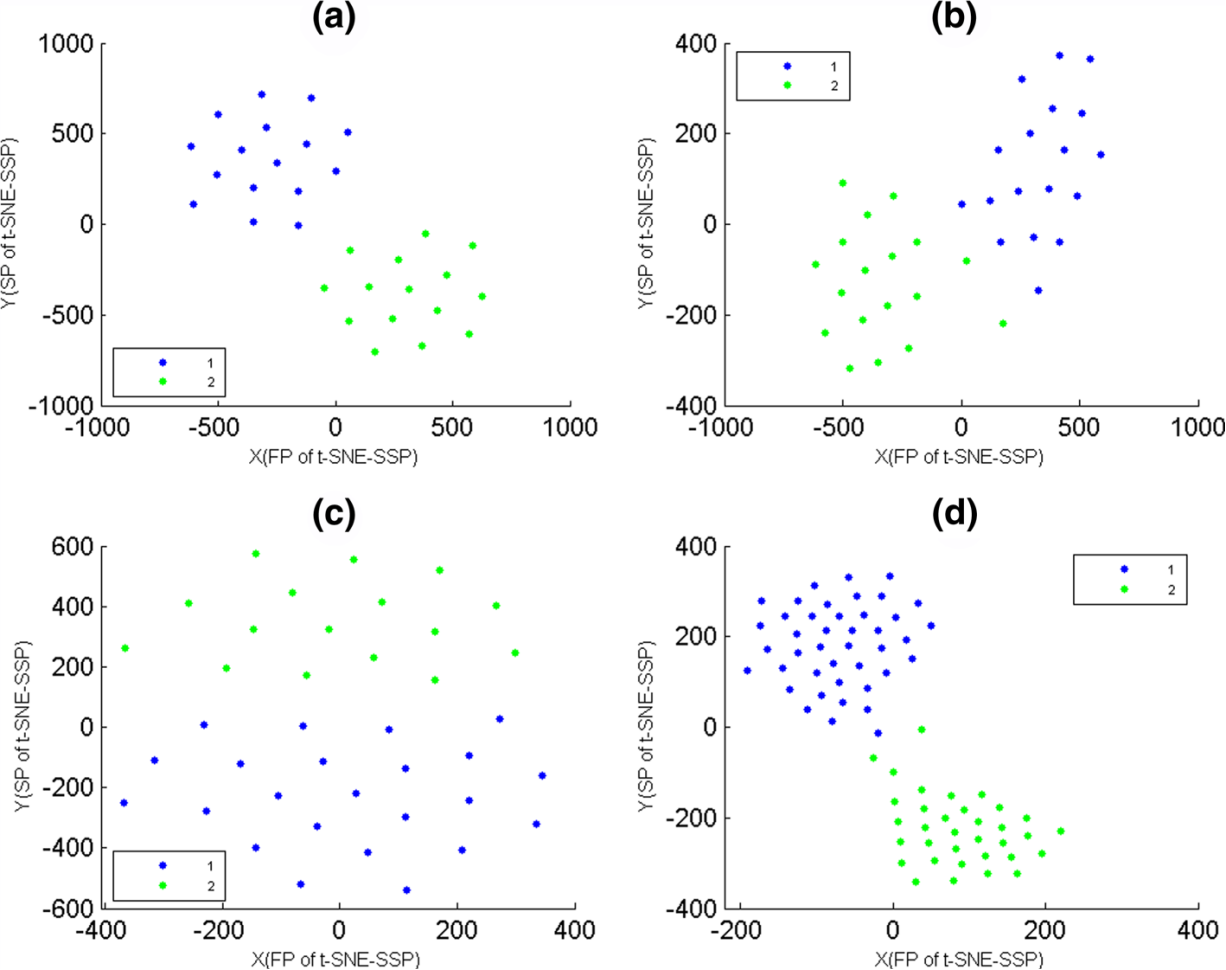
The t-SNE-SSP maps of tumor and normal samples of data-1, 2, 3 and 4. The samples were colored according to their population membership. The X-axis represented the first projections(FP) of t-SNE-SSP. The Y-axis represented the second projections(SP) of t-SNE-SSP. **a** The t-SNE-SSP map of tumor and normal samples of data-1. **b** The t-SNE-SSP map of tumor and normal samples of data-2. **c** The t-SNE-SSP map of tumor and normal samples of data-3. **d** The t-SNE-SSP map of tumor and normal samples of data-3

Here, we used data-1, 2, 3 and 4 to assess the biological reliability of t-SNE-SSP maps. According to population membership of samples, these four data sets were mapped on t-SNE-SSP maps respectively (Fig. 3), where t-SNE-SSP maps of data-1, 2, 3 and 4 were t-SNE-SS(20), t-SNE-SS(19), t-SNE-SS(30) and t-SNE-SS(18), respectively. In fact, for tumor and normal samples of different populations, their biological partitioning were not always obvious from those differentially expressed genes, but Fig. 3 clearly showed that t-SNE-SSP maps were able to project samples of the same populations into the same together, which could help us to understand the relationships between different populations.

### The consistency between MG-PCC algorithm and t-SNE-SSP maps

**Fig. 4:**
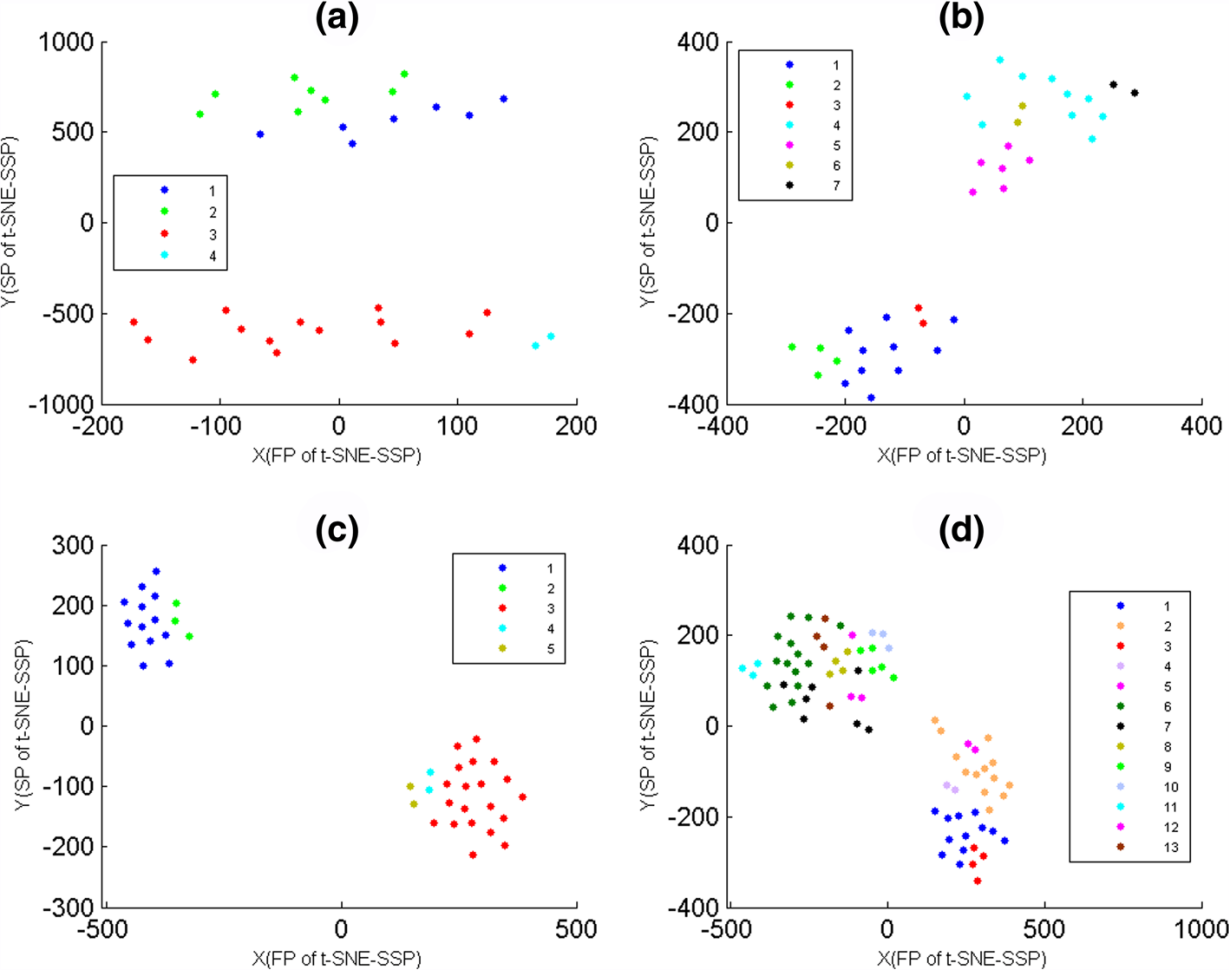
The t-SNE-SSP maps of mini-groups of data-1, 2, 3 and 4. These mini-groups were generated from MG-PCC algorithm, where samples were colored according to their mini-group memberships. The X-axis represented the first projections(FP) of t-SNE-SSP. The Y-axis represented the second projections(SP) of t-SNE-SSP. **a** The t-SNE-SSP map of 4 mini-groups of data-1. **b** The t-SNE-SSP map of 7 mini-groups of data-2. **c** The t-SNE-SSP map of 5 mini-groups of data-3. **d** The t-SNE-SSP map of 13 mini-groups of data-4

Here, for mini-groups of data-1, 2, 3 and 4, they were used to assess the consistency between MG-PCC algorithm and t-SNE-SSP maps, where and data-1, 2, 3 and 4 were divided into 4, 7, 5 and 13 mini-groups by MG-PCC algorithm, respectively. According to mini-group membership of samples, these four data sets were mapped on t-SNE-SSP maps (Fig. 4), where t-SNE-SSP maps of data-1, 2, 3 and 4 were t-SNE-SS(6), t-SNE-SS(8), t-SNE-SS(12) and t-SNE-SS(12), respectively. From Fig. 4(a), (b) and (c), t-SNE-SSP maps of data-1, 2, and 3 were able to project samples of the same mini-groups together. But for mini-groups of data-4, the seventh mini-group had slightly intermixing with others (Fig. 4(d)), where the samples of the seventh mini-group were marked by black points. In fact, for 23 mini-groups of data-5 that were generated from MG-PCC algorithm, their relationships were not obvious displayed by their t-SNE-SSP map also. That is, the exhibition effects of t-SNE-SSP maps might weaken when the number of mini-groups was relatively large.

### Comparison of t-SNE-SSP and PCA-S

**Fig. 5:**
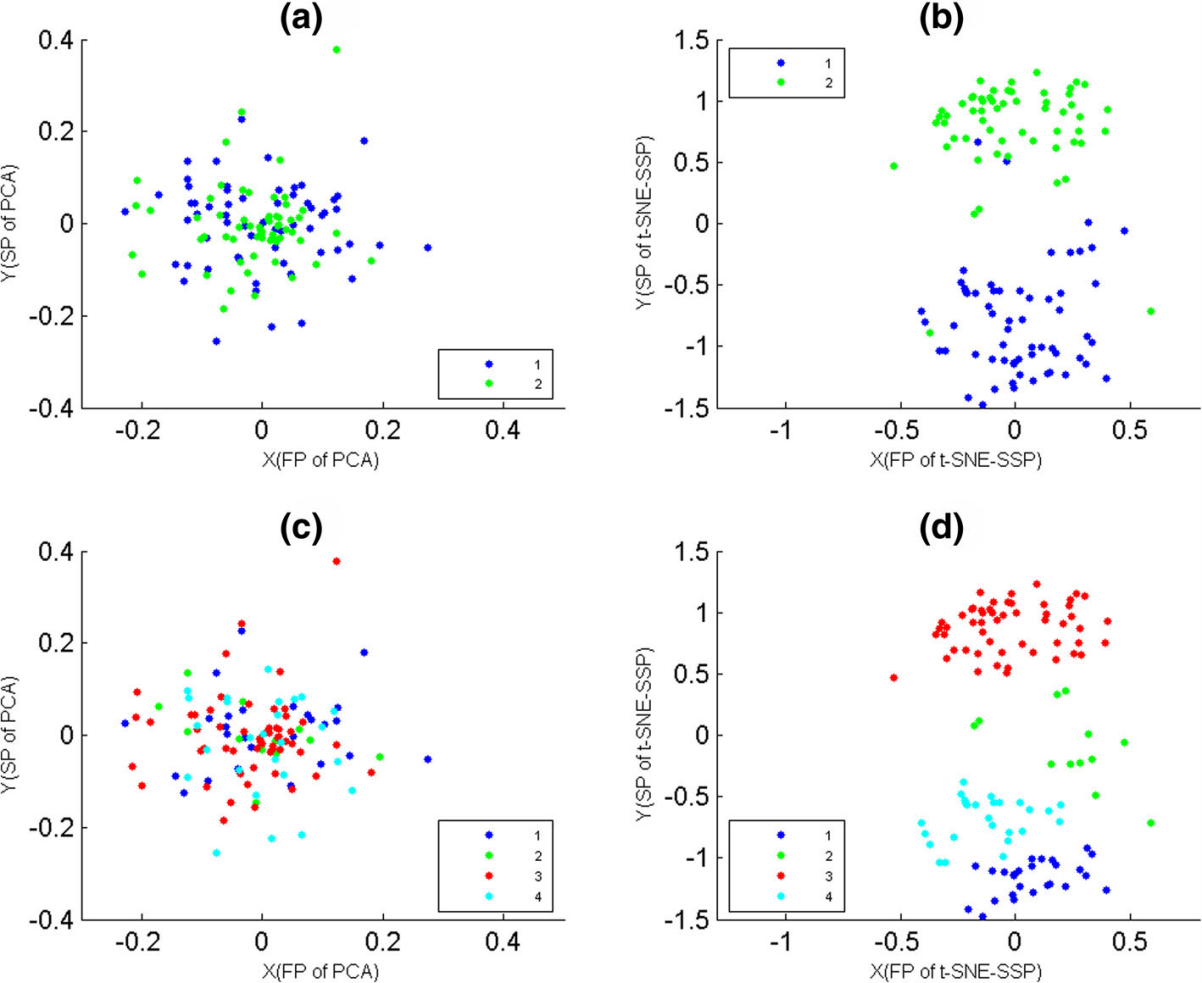
The t-SNE-SSP and PCA-S maps of data-5. The X-axis represented the first projections(FP) of t-SNE-SSP(or PCA-S). The Y-axis represented the second projections(SP) of t-SNE-SSP(or PCA-S). The samples were colored according to their cluster membership. **a** PCA-S map of tumor and normal samples. **b** The t-SNE-SSP map of tumor and normal samples. **c** PCA-S map of 4 clusters, where clusters were generated from K-means with PCC. **d** The t-SNE-SSP map of 4 clusters, where clusters were generated from K-means with PCC

Here, for tumor and normal samples of data-5, they were mapped on PCA-S and t-SNE-SSP maps (Fig. 5 (a) and (b)) according to their population memberships, where PCA-S is PCA of samples, and t-SNE-SSP map of data-5 were t-SNE-SS(18). Then, samples of data-5 were divided into 4 clusters by K-means with PCC, and the clustering result was overlaid on PCA-S and t-SNE-SSP(t-SNE-SS(19)) maps (Fig. 5 (c) and (d)) also. For biological classifications and PCC clusters of data-10, Fig. 5 showed that t-SNE-SSP maps provided them good 2D projections (Fig. 5 (b) and (d)), but PCA-S maps had significant intermixing for them (Fig. 5 (a) and (c)). For biological classifications and PCC clusters of other data sets in this paper, PCA-S gave them poor visualization also.

In fact, for the optimization criterion of PCA, the relationship of distant points was able to depict as accurately as possible, while small inter-point distances might be distorted [14]. Moreover, there might be no single linear projection that gave a good view for most gene expression data [14]. Thus, for complex gene expression data sets, many linear projection methods might fail.

### The reliability of t-SNE-SGI maps

**Fig. 6:**
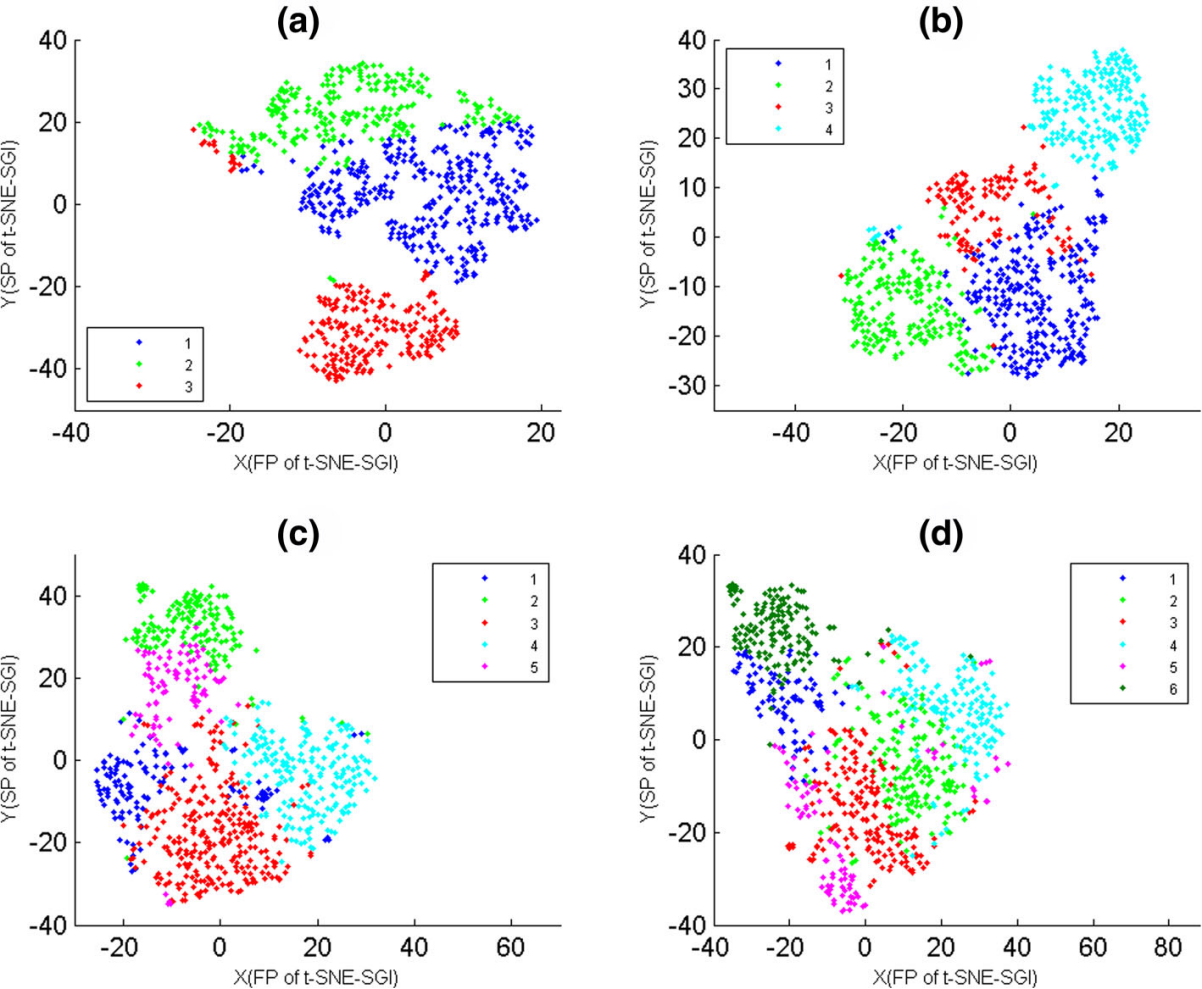
The t-SNE-SGI maps of data-9. The genes of data-9 were divided into 3, 4, 5 and 6 clusters by K-means with PCC, where genes were colored according to their cluster membership. The X-axis represented the first projections(FP) of t-SNE-SGI. The Y-axis represented the second projections(SP) of t-SNE-SGI. **a** The t-SNE-SGI map of 3 clusters. **b** The t-SNE-SGI map of 4 clusters. **c** The t-SNE-SGI map of 5 clusters. **d** The t-SNE-SGI map of 6 clusters

For 3, 4, 5 and 6 clusters of data-9 that were generated from K-means with PCC, they were shown on t-SNE-SGI maps (Fig. 6), where t-SNE-SGI maps of 3, 4, 5 and 6 clusters were t-SNE-SG(4), t-SNE-SG(5), t-SNE-SG(8) and t-SNE-SG(9), respectively. Figure 6 showed that t-SNE-SGI gave the relatively clear 2D projections for 3, 4 and 5 clusters, but had significant intermixing for 6 clusters. That is, t-SNE-SGI maps might weaken when the number of clusters was relatively large.

Compared to K-means clustering analysis, MG-PCC algorithm does not estimate the number of clusters. But for genes, MG-PCC algorithm generates a large number of mini-groups, which can make genes with the similar biological function into different mini-groups. Thus, MG-PCC algorithm is not appropriate to cluster genes.

### Comparison of t-SNE-SGI, t-SNE-N and t-SNE-O maps

**Fig. 7:**
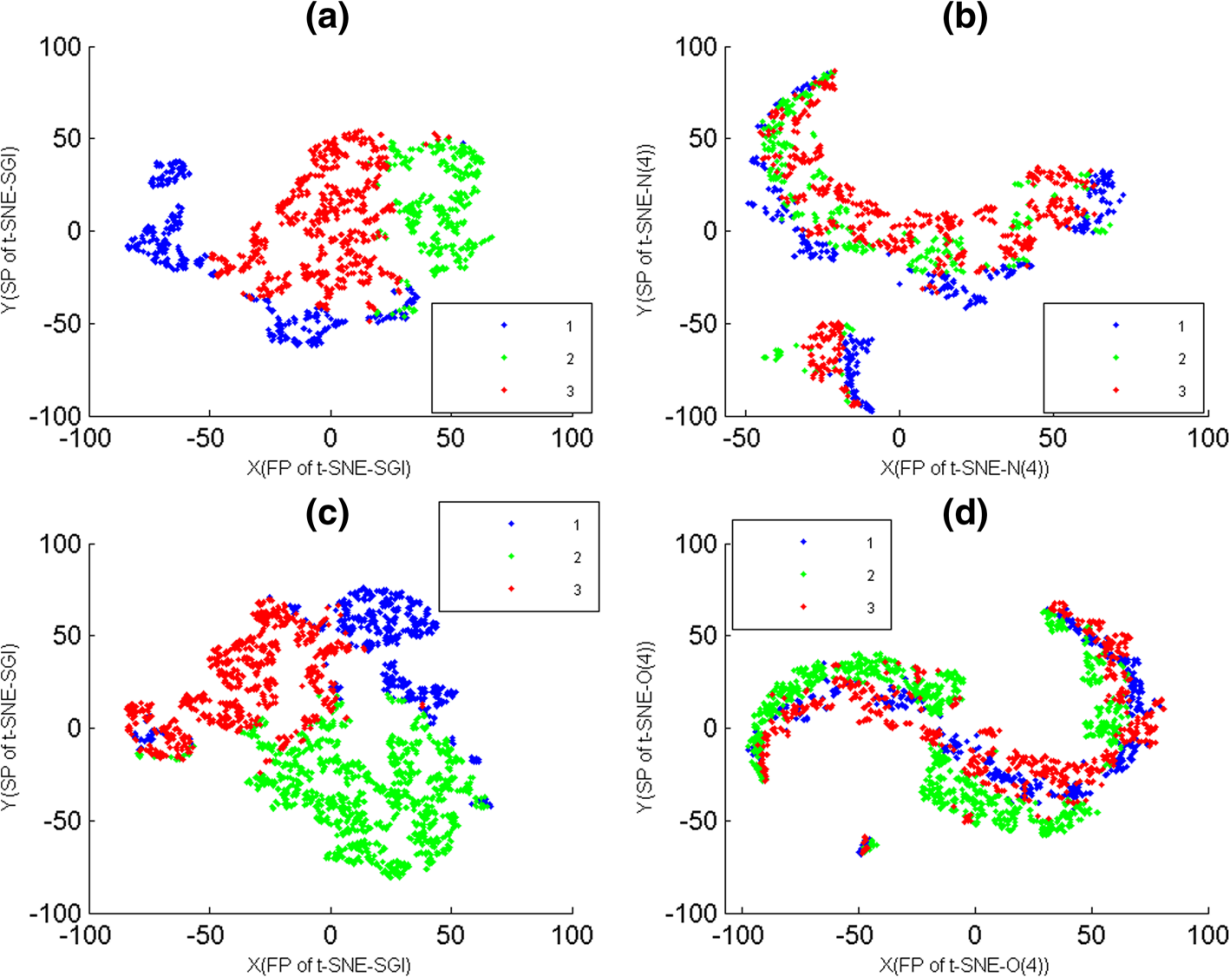
The t-SNE-SGI, t-SNE-N and t-SNE-O maps of data-7 and data-8. The genes of data-7 and data-8 were divided into 3 clusters by K-means with PCC, where genes were colored according to cluster membership. The X-axis represented the first projections(FP) of t-SNE. The Y-axis represented the second projections(SP) of t-SNE. **a** The t-SNE-SGI map of 3 clusters of data-7. **b** The t-SNE-N map of 3 clusters of data-7. **c** The t-SNE-SGI map of 3 clusters of data-8. **d** The t-SNE-O map of 3 clusters of data-8

Here, O-genes data-7 and data-8 were firstly divided into 3 clusters by K-means with PCC, and then these clustering results were overlaid on t-SNE-SGI, t-SNE-N and t-SNE-O maps (Fig. 7), where t-SNE-N and t-SNE-O maps were t-SNE maps of O-genes and N-genes respectively, and their initialization dimensions were the same as t-SNE-SGI. Figure 7 showed that t-SNE-SGI provided these clustering results good 2D projections, but t-SNE-N and t-SNE-O maps had significant intermixing.

For PCC clusters of data-6, 9 and 10, when t-SNE-N and t-SNE-O maps gave them poor visualizations also. The reason was that PCC and Euclidean clusters of O-genes and N-genes had significant differences.

### Constructing the nearest sample neighbor map by t-SNE-SS(m)

**Fig. 8:**
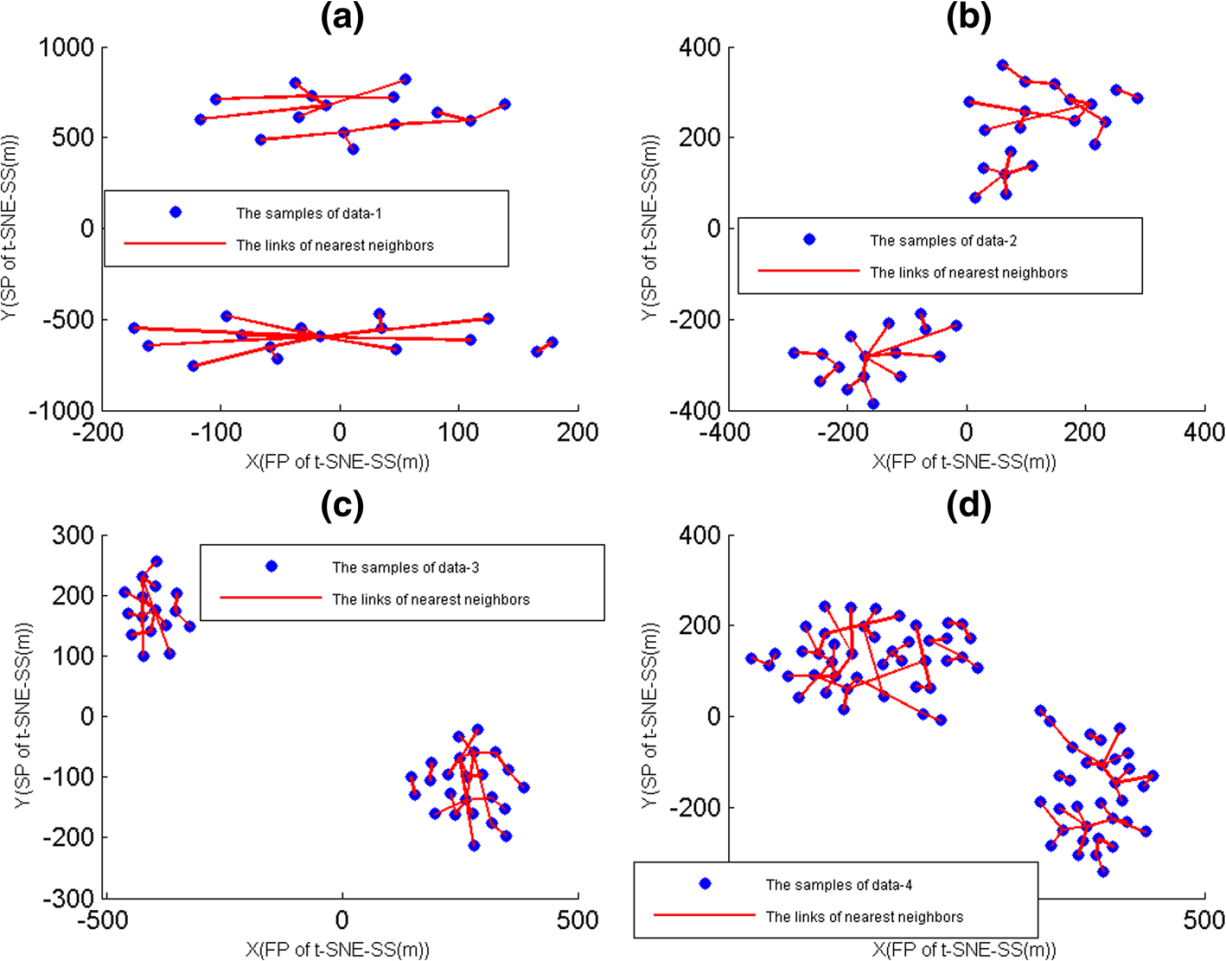
The nearest sample neighbors maps. The X-axis represented the first projections(FP) of t-SNE-SS(m). The Y-axis represented the second projections(SP) of t-SNE-SS(m). The nearest gene neighbor were lined by red line. **a** The nearest sample neighbors of data-1. **b**The nearest sample neighbors of data-2. **c** The nearest sample neighbors of data-3. **d** The nearest sample neighbors of data-4

For gene expression data sets under samples, the hierarchical clustering were used to display their sample neighbors usually [27], but the method was likely to cause loose sample neighbors. By D-plots [19], t-SNE-SS(m) maps were able to generate more valid gene neighbors compared to t-SNE-SSP, where *m* was the dimension of samples. Here, we constructed of the nearest sample neighbors by t-SNE-SS(m) map, where sample neighbors were defined by PCC. For sample neighbors of data-1, 2, 3 and 4, they were displayed on Fig. 8, where the nearest gene neighbor were lined by red line.

Figure 8 showed that sample neighbors had created several independent tree diagrams. In fact, each tree diagram was corresponding to a mini-group of samples. Thus, the combination of t-SNE-SS(m) map and MG-PCC algorithm was able to help us to search subtypes of samples.

### Constructing the nearest gene neighbor map by t-SNE-SG(n)

**Fig. 9:**
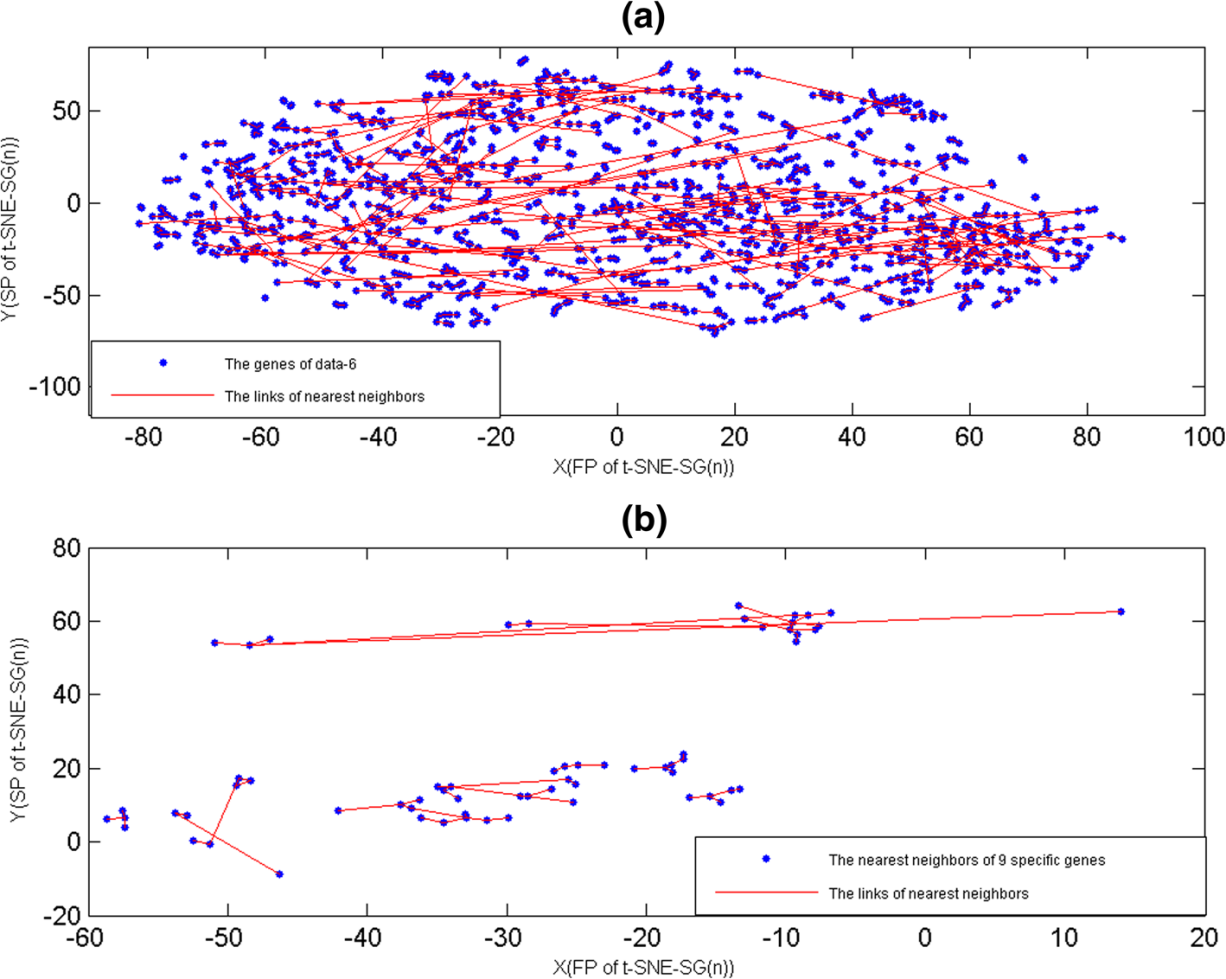
The nearest gen neighbor maps. The X-axis represented the first projections(FP) of t-SNE-SG(n). The Y-axis represented the second projections(SP) of t-SNE-SG(n). The nearest gene neighbor were lined by red line. **a** The nearest gene neighbors of data-6. **b**The nearest neighbors of 9 specific genes that were smoking independent

In fact, for t-SNE method that had used to construct gene neighbors, where the initialization dimension of these t-SNE was dimension of genes [14, 20]. Here, we constructed of the nearest gene neighbors by t-SNE-SG(n), where gene neighbors were defined by PCC, and we focused our attention on data-6. For gene neighbors of data-1, they were displayed on Fig. 9(a). From Fig. 9(a), gene neighbors had created many independent tree diagrams also, and these tree diagrams were corresponding to mini-groups that were generated from MG-PCC algorithm.

Based on GDS3837, GDS3257 and GDS3054, nine differentially expressed genes that were associated with lung cancer had been extracted, where these 9 genes that were smoking independent, and they were AGER, CA4, EDNRB, FAM107A, GPM6A, NPR1, PECAM1, RASIP1 and TGFBR3 [16]. Here, we used t-SNE-SG(n) map to display these nine mini-groups that contained nine specific genes (Fig. 9(b)). From Fig. 9(b), these nine independent tree diagrams might help us to search correlation genes.

## Discussion

For samples of gene expression data sets of cancers, there are no clear boundary between subtypes of samples usually [7]. The reason is that the high dimensions of samples often results in the different subtypes to be isometric [9]. Here, we use MG-PCC algorithm to divide samples into mini-groups, and results show that the algorithm can put tumor and normal samples into their respective mini-groups. In fact, MG-PCC algorithm puts the nearest neighbors in the same mini-groups, which can distinguish the inconspicuous differences of different subtypes of samples. However, when MG-PCC algorithm applies genes, it generates a large number of mini-groups. That is, for genes with similar expression patterns, they may be put to different mini-groups, which make difficult to group genes with the similar biological function together. The reason is that MG-PCC algorithm does not presuppose the number of mini-groups, and the similar genes are not necessarily the nearest neighbors. Moreover, for the large number of mini-groups, any dimension reduction technique may give messy visualizations for the entire data set. Thus, MG-PCC algorithm is not appropriate to divide genes.

To efficiently display mini-groups of samples that are generated from MG-PCC algorithm, we firstly verify that PCC and Euclidean clusters of the standardized samples are more consistent compared to the original and normalized ones, and PCC of the standardized samples are the same as the original and normalized ones. Since t-SNE maps have been successful in displaying clusters of Euclidean distance, t-SNE maps of the standardized samples can give good visualizations for mini-groups also. However, for t-SNE maps of the standardized samples, they have significant difference for different parameters, and most of them give poor visualizations for mini-groups also. To select the optimal t-SNE maps of mini-groups, t-SNE-SSP are constructed secondly, where t-SNE-SSP maps are selected from these t-SNE maps of the standardized samples with different perplexity parameter. Results show that that t-SNE-SSP maps give mini-groups of samples good visualizations, and give PCC clusters of samples good visualizations also. However, for t-SNE-SSP maps, when we use them to display PCC clusters of genes, they give fuzzy visualizations. The reason may be that the dimensions of samples are far more than ones of genes. To efficiently map PCC clusters of genes, t-SNE-SGI maps are constructed, where t-SNE-SGI maps are selected from these t-SNE maps of the standardized genes with different initialization dimensions. By several gene expression data sets of cancers, we verify that SNE-SGI maps can give PCC clusters of genes good visualizations. Furthermore, we use t-SNE-SS(m) and t-SNE-SG(n) maps to display the nearest neighbor of samples and genes respectively, which make the relationships between samples(or genes) easy to visualize and understand. In total, for gene expression data sets of cancers, these four types of t-SNE maps identify them easy and intuitive.

## Conclusion

In this article, we use MG-PCC algorithm to divide samples of gene expression data sets into mini-groups, and t-SNE-SSP to display the relationships of these mini-groups. Moreover, we provide t-SNE-SGI maps to display PCC clusters of genes, and t-SNE-SS(m) and t-SNE-SG(n) maps to display the nearest neighbor of samples and genes respectively. In total, for MG-PCC algorithm and these four types of t-SNE maps, they can help us to understand the entire gene expression data sets when they coordinate with each other.

## Additional file

Additional file 1 MATLAB algorithm. A freely available MATLAB implemented to perform MG-PCC, t-SNE-SS, t-SNE-SG and draw the nearest sample(or gene) neighbors for a data set. (ZIP 6873 kb)

## Abbreviations

2D: two dimensional
A-value: the quantifying criterion of the projecting maps
MG-PCC: the algorithm using PCC to put the nearest neighbors into the same mini-groups
N-genes: the normalized gene
O-genes: the original gene
PCA: the principal component analysis
PCA-S: PCA of the standardized samples
PCC: Person correlation coefficient
S-genes: the standardized genes
the standardized samples: the standardized samples
t-SNE: t-statistic Stochastic Neighbor Embedding
t-SNE-N: t-SNE of N-genes
t-SNE-O: t-SNE of O-genes
t-SNE-SG: t-SNE of standardized genes
t-SNE-SGI: t-SNE-SG that its A-value is the smallest
t-SNE-SS: t-SNE of standardized samples
t-SNE-SSP: t-SNE-SG that its A-value is the smallest
