# Supplementary figures and images for: Analyzing the similarity of samples and genes by MG-PCC algorithm, t-SNE-SS and t-SNE-SG maps
Source: BMC Bioinformatics. 2018 Dec 17;19:512. doi: 10.1186/s12859-018-2495-5 (PMC6296107; doi:10.1186/s12859-018-2495-5)

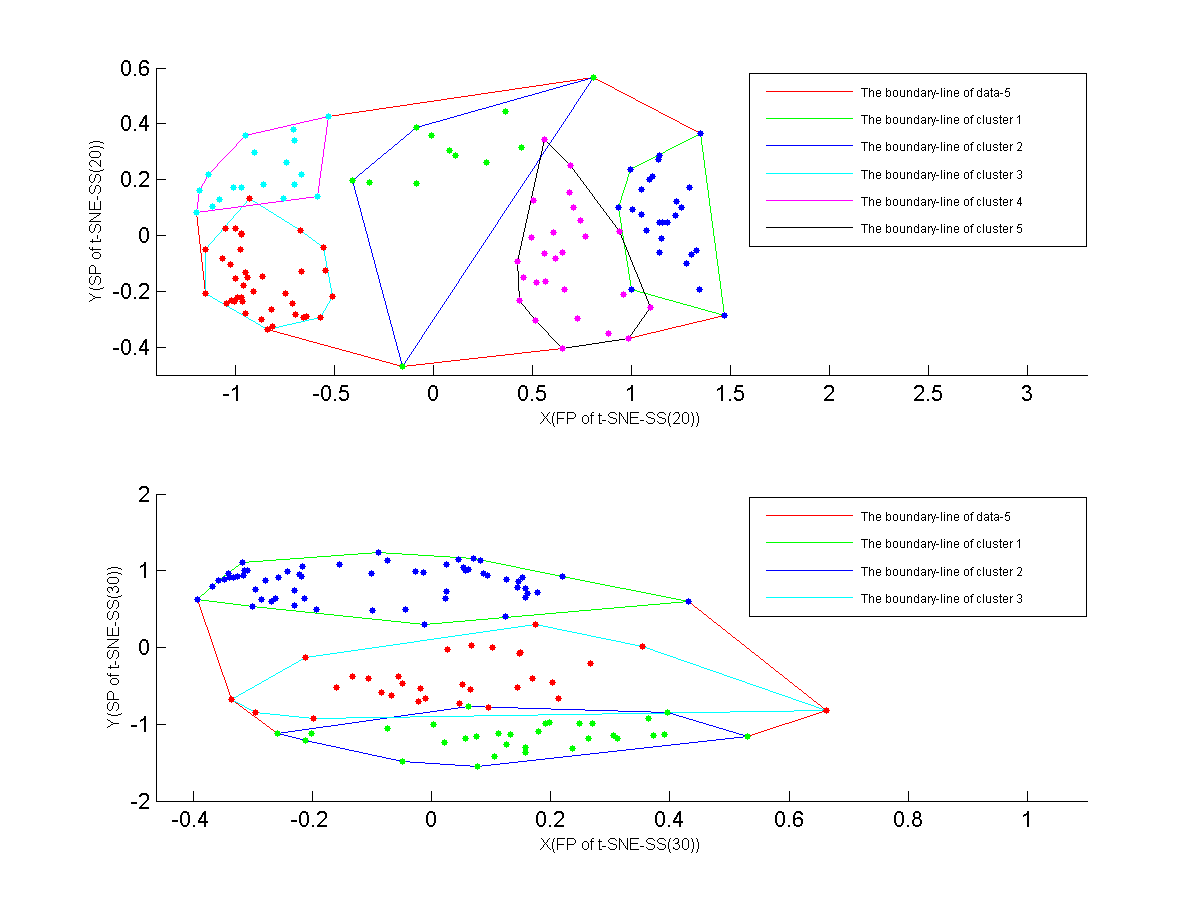

Supplement: Supplementary file 1 — MATLAB algorithm. A freely available MATLAB implemented to perform MG-PCC, t-SNE-SS, t-SNE-SG and draw the nearest sample(or gene) neighbors for a data set. (ZIP 6873 kb) [file 12859_2018_2495_MOESM1_ESM.zip › Fig 1.tif]

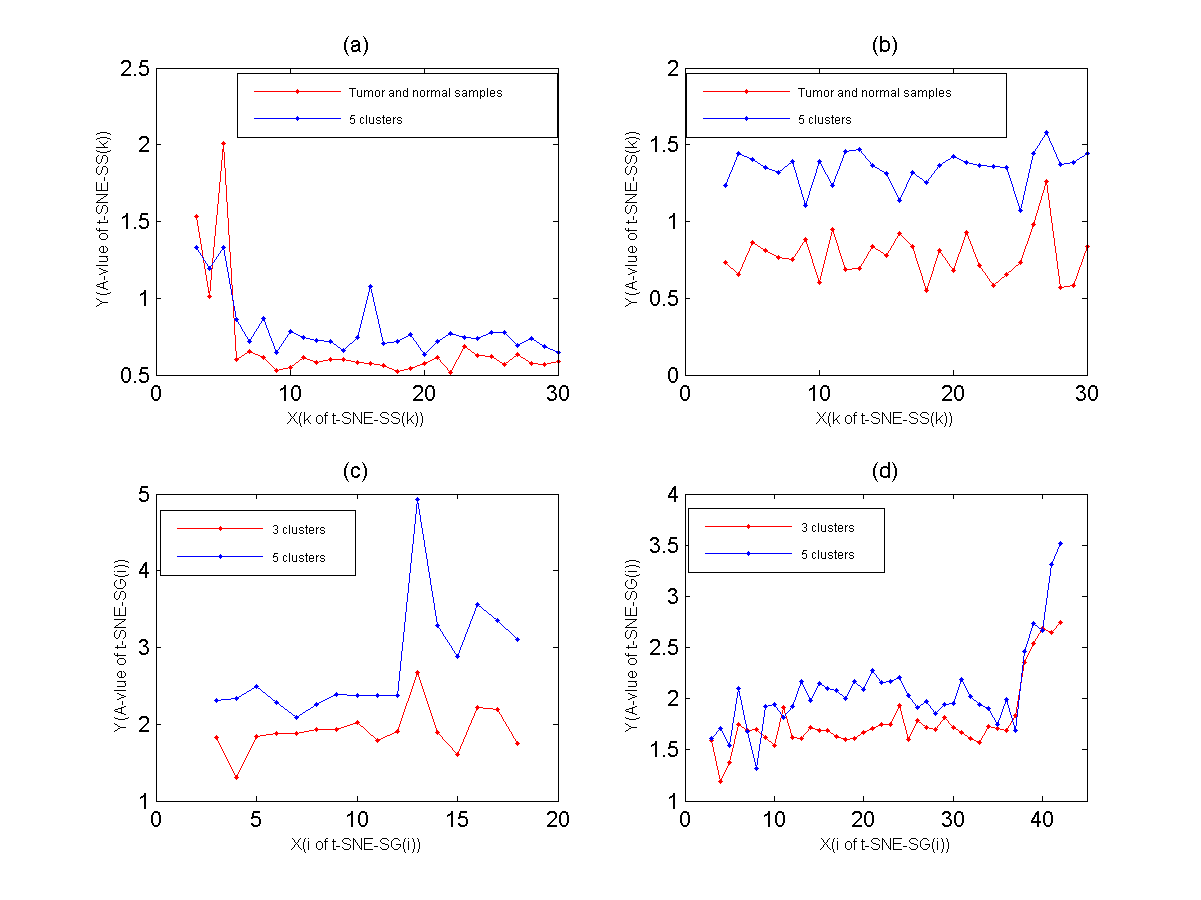

Supplement: Supplementary file 1 — MATLAB algorithm. A freely available MATLAB implemented to perform MG-PCC, t-SNE-SS, t-SNE-SG and draw the nearest sample(or gene) neighbors for a data set. (ZIP 6873 kb) [file 12859_2018_2495_MOESM1_ESM.zip › Fig 2.tif]

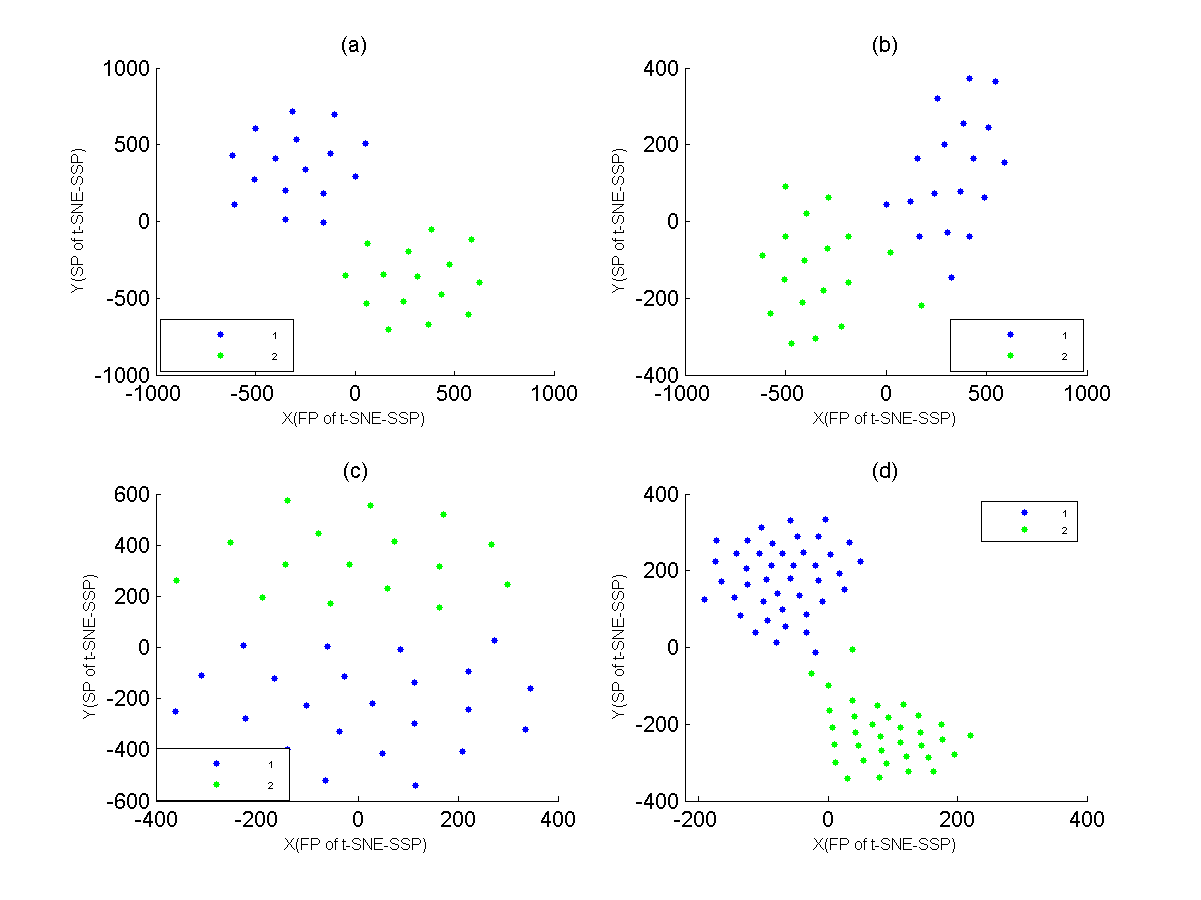

Supplement: Supplementary file 1 — MATLAB algorithm. A freely available MATLAB implemented to perform MG-PCC, t-SNE-SS, t-SNE-SG and draw the nearest sample(or gene) neighbors for a data set. (ZIP 6873 kb) [file 12859_2018_2495_MOESM1_ESM.zip › Fig 3.tif]

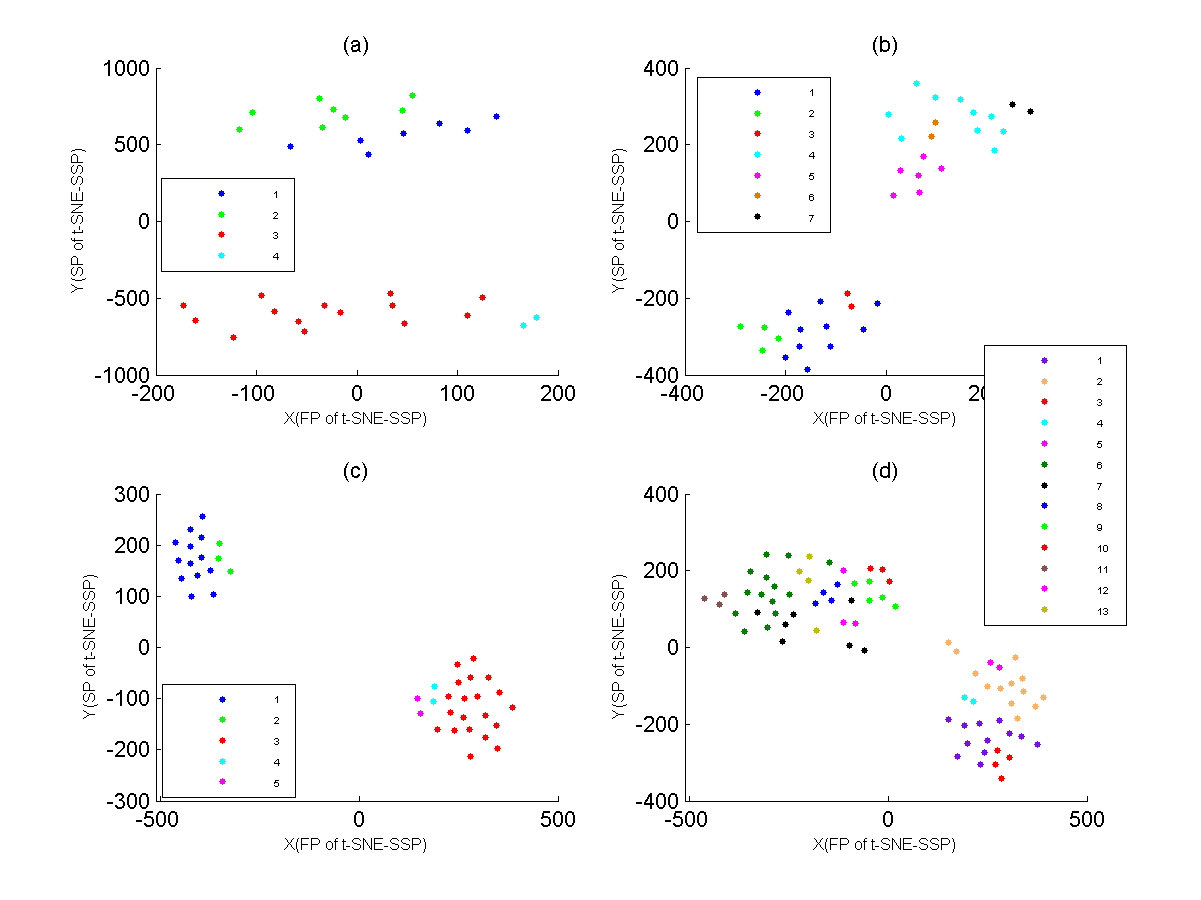

Supplement: Supplementary file 1 — MATLAB algorithm. A freely available MATLAB implemented to perform MG-PCC, t-SNE-SS, t-SNE-SG and draw the nearest sample(or gene) neighbors for a data set. (ZIP 6873 kb) [file 12859_2018_2495_MOESM1_ESM.zip › Fig 4.tif]

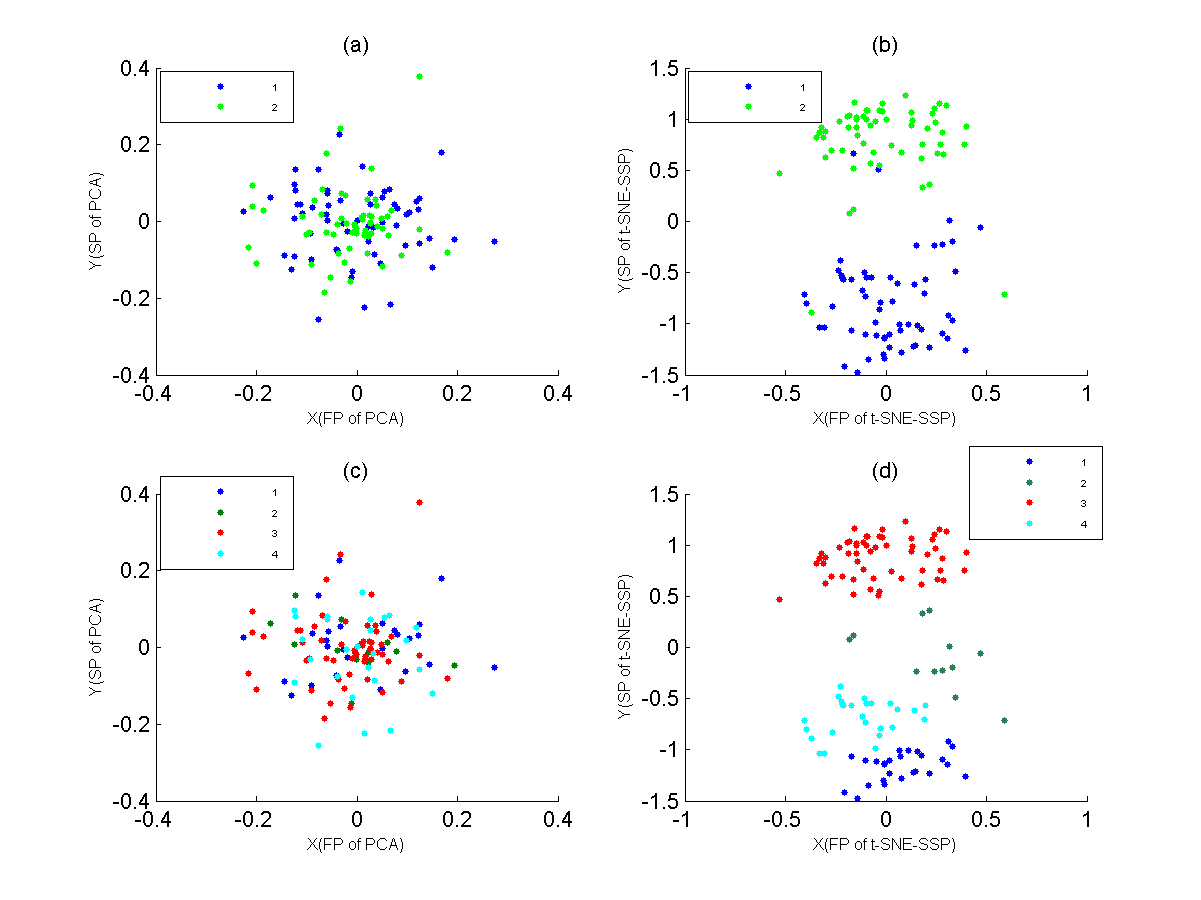

Supplement: Supplementary file 1 — MATLAB algorithm. A freely available MATLAB implemented to perform MG-PCC, t-SNE-SS, t-SNE-SG and draw the nearest sample(or gene) neighbors for a data set. (ZIP 6873 kb) [file 12859_2018_2495_MOESM1_ESM.zip › Fig 5.tif]

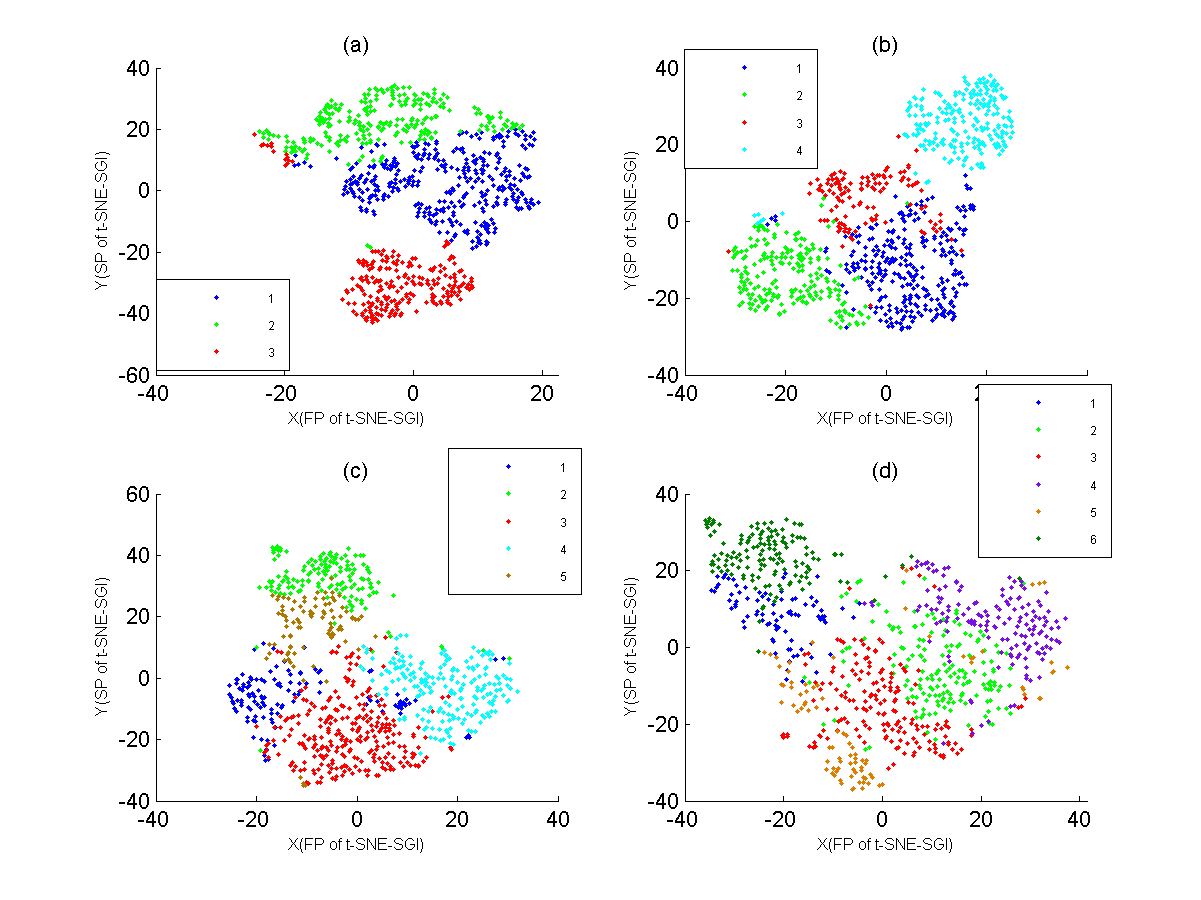

Supplement: Supplementary file 1 — MATLAB algorithm. A freely available MATLAB implemented to perform MG-PCC, t-SNE-SS, t-SNE-SG and draw the nearest sample(or gene) neighbors for a data set. (ZIP 6873 kb) [file 12859_2018_2495_MOESM1_ESM.zip › Fig 6.tif]

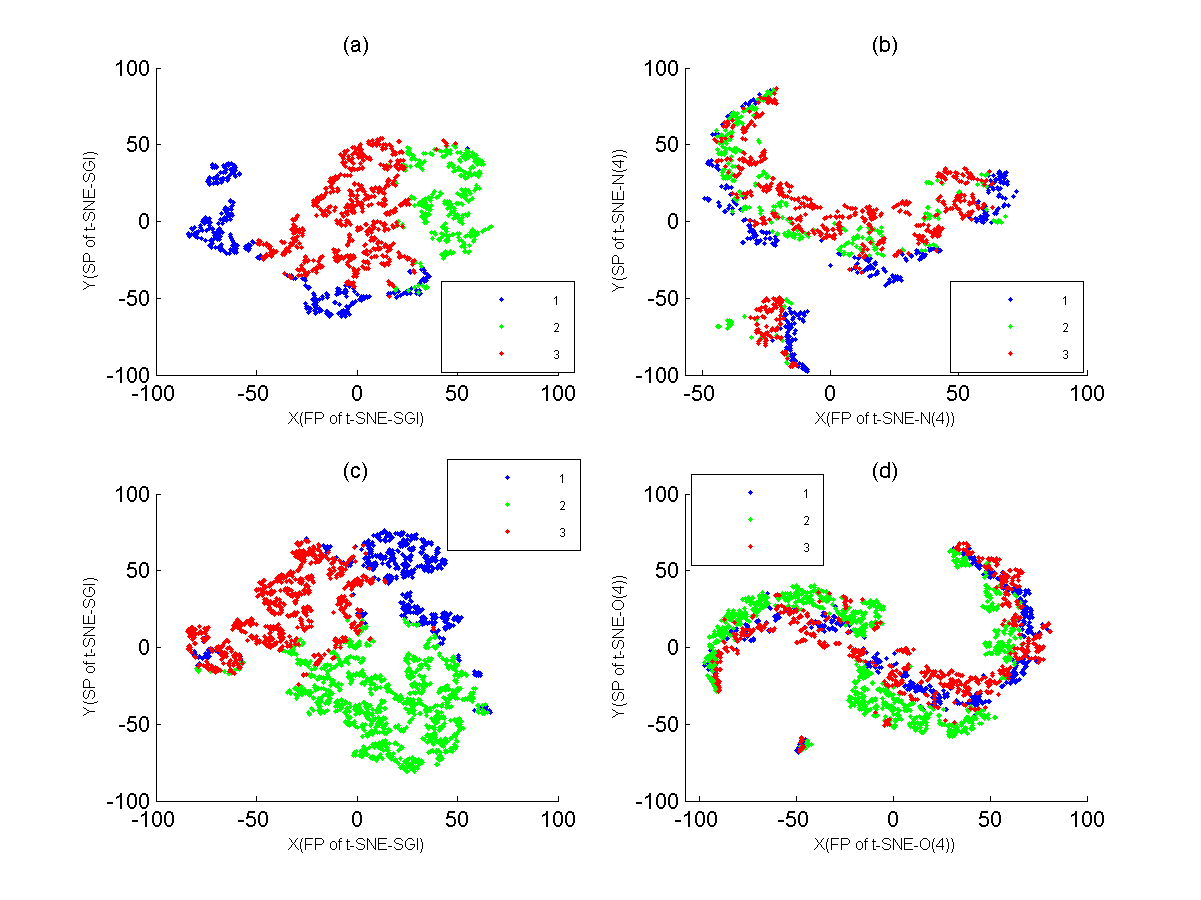

Supplement: Supplementary file 1 — MATLAB algorithm. A freely available MATLAB implemented to perform MG-PCC, t-SNE-SS, t-SNE-SG and draw the nearest sample(or gene) neighbors for a data set. (ZIP 6873 kb) [file 12859_2018_2495_MOESM1_ESM.zip › Fig 7.tif]

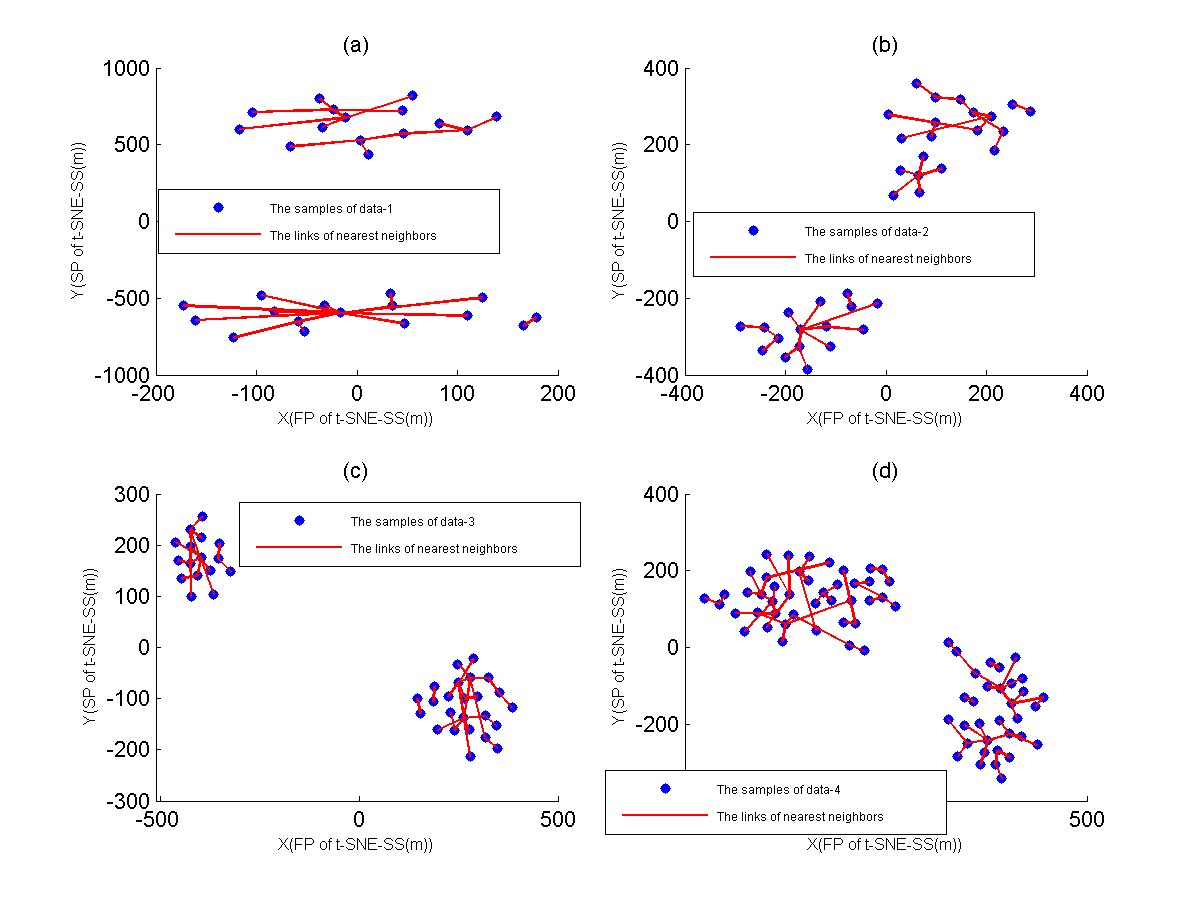

Supplement: Supplementary file 1 — MATLAB algorithm. A freely available MATLAB implemented to perform MG-PCC, t-SNE-SS, t-SNE-SG and draw the nearest sample(or gene) neighbors for a data set. (ZIP 6873 kb) [file 12859_2018_2495_MOESM1_ESM.zip › Fig 8.tif]

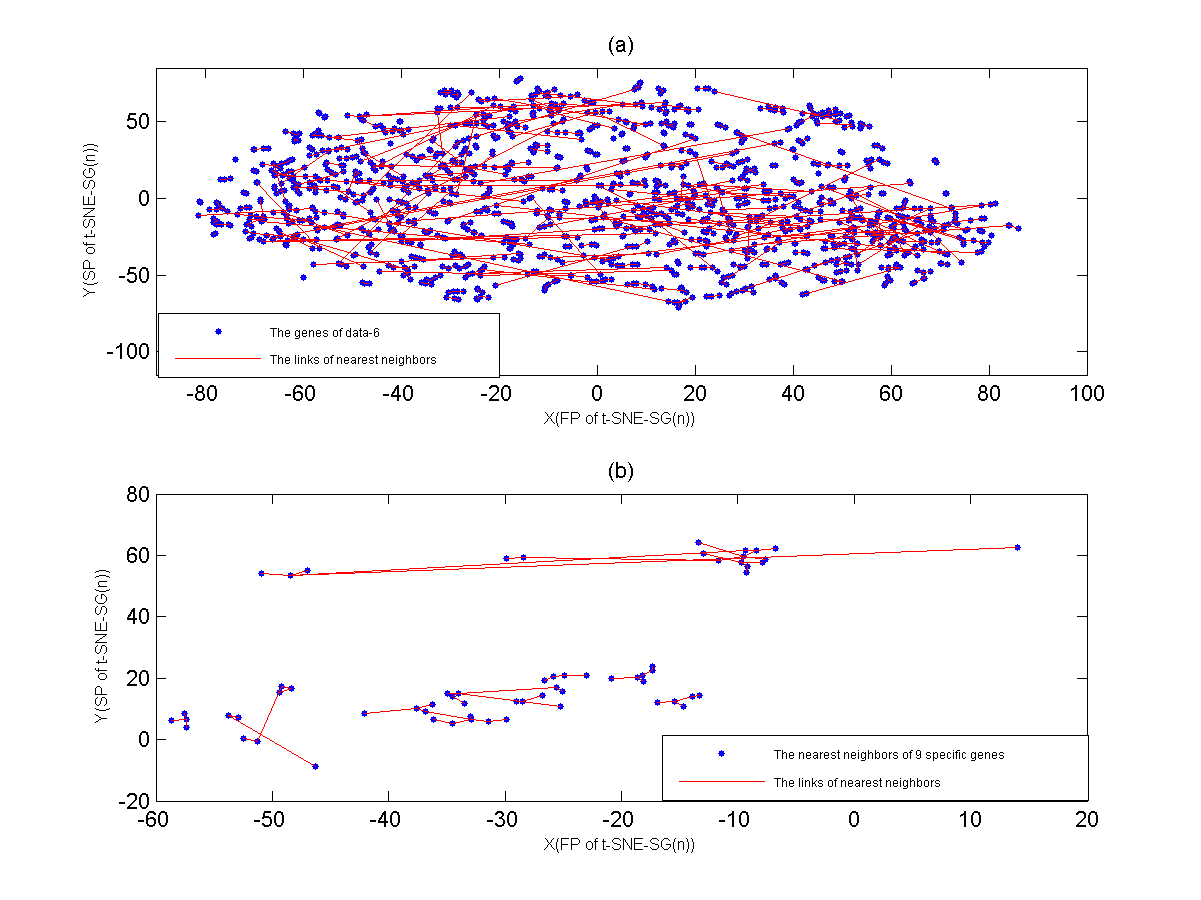

Supplement: Supplementary file 1 — MATLAB algorithm. A freely available MATLAB implemented to perform MG-PCC, t-SNE-SS, t-SNE-SG and draw the nearest sample(or gene) neighbors for a data set. (ZIP 6873 kb) [file 12859_2018_2495_MOESM1_ESM.zip › Fig 9.tif]
